# West Nile virus encephalitis in GATA2 deficiency

**DOI:** 10.1186/s13223-019-0321-x

**Published:** 2019-01-24

**Authors:** Jaime S. Rosa, Shanthi Kappagoda, Amy P. Hsu, Joie Davis, Steven M. Holland, Anne Y. Liu

**Affiliations:** 1 Division of Allergy, Immunology, and Rheumatology, Department of Pediatrics, Stanford University School of Medicine, 269 Campus Drive, CCSR 3215, MC 5366, Stanford, CA 94305 USA; 2 Division of Infectious Diseases and Geographic Medicine, Department of Medicine, Stanford University School of Medicine, Stanford, CA 94305 USA; 3 Laboratory of Clinical Immunology and Microbiology, National Institute of Allergy and Infectious Diseases, National Institutes of Health, Bethesda, MD 20892 USA

**Keywords:** West Nile virus, Encephalitis, GATA2, Interferon, Immunoglobulin

## Abstract

We report a male with longstanding warts who presented with severe West Nile virus encephalitis (WNVE) and recovered after interferon alfa-2b and intravenous immunoglobulin. He was later found to have GATA2 deficiency and underwent successful hematopoietic stem cell transplant.

## Introduction

A 24-year-old Caucasian male beekeeper was admitted for altered mental status. Within the past 2 weeks during the month of August, he had been camping in Wyoming, where he and his companions suffered from mosquito bites. All of them had rashes that resolved after 3 days, but our patient subsequently developed worsening fevers, headache, confusion, abdominal pain, vomiting, and diarrhea. MRI of the brain showed T2-signal hyperintensities within the deep white matter. Electroencephalography was normal but lumbar puncture revealed pleocytosis, consistent with CNS infection (Table 1). He was started on vancomycin, ceftriaxone, doxycycline, acyclovir, and dexamethasone empirically and was transferred to the intensive care unit. This patient was agitated and unable to follow instructions. CSF West Nile virus (WNV) IgM and serum WNV IgM and RNA were detectable while the remainder of the infectious workup was negative. Peripheral blood flow cytometry demonstrated pancytopenia with complete absence of monocytes (Table 1); lymphocytes were markedly decreased, including CD16+56+natural killer (NK) cells and CD56^bright^CD16+NK cells. NK cell cytotoxicity assay was normal.

**Table 1:** Laboratory testing results at different time periods of this patient’s presentation

Laboratory testing	Six years prior to WNVE presentation	Current presentation	Normal ranges
Peripheral blood and serum			
White blood cells (cells/*μ*L)	1800	2100	(4500–13,000)
Neutrophils (cells/*μ*L)	1062	1810	(1800–8000)
Lymphocytes (cells/*μ*L)	702	270	(1200–5200)
Monocytes (cells/*μ*L)	18	0	(200–900)
Eosinophils (cells/*μ*L)	18	10	(15–500)
Basophils (cells/*μ*L)	18	0	(0–200)
Platelets (cells/*μ*L)	184,000	103,000	(140,000–400,000)
Hemoglobin (g/L)	12.7	11.3	(12.0–16.9)
Hematocrit (%)	36.8	32.0	(36.0–49.0)
CD3 T lymphocytes (cells/*μ*L)	NM	288	(700–2100)
CD4 T lymphocytes (cells/*μ*L)	NM	161	(300–1400)
CD8 T lymphocytes (cells/*μ*L)	NM	68	(200–900)
CD56+16 NK lymphocytes (cells/*μ*L)	NM	13	(90–600)
CD19 B lymphocytes (cells/*μ*L)	NM	8	(100–500)
IgG	3100	2180	(694–1618)
IgG_1_	2340	1780	(382–929)
IgG_2_	397	203	(241–700)
IgG_3_	323	140	(22–178)
IgG_4_	2.2	2.2	(4–48)
IgA	329	217	(81–463)
IgM	101	66	(48–271)
Cerebrospinal fluid			
CSF: White blood cells (cells/*μ*L)	NM	8	(0–5)
CSF: Polymorphonuclear cells (cells/*μ*L)	NM	5	(0)
CSF: Mononuclear cells (cells/*μ*L)	NM	3	(0–5)
CSF: Red blood cells (cells/*μ*L)	NM	3	(0–10)

*HPF* high power field, *NM* not measured

The patient’s childhood history included rosacea and intermittent left foot swelling occurring once a year. Beginning age 7, he developed warts on his hands, feet, digits, and limbs that recurred despite multiple attempts at removal. He suffered multiple episodes of pneumonia, bronchitis, and sinusitis. At age 18, he was referred to a hematologist for persistent leukopenia and intermittent thrombocytopenia (Table 1). Bone marrow aspirate and biopsy showed reduced myeloid precursors, with marked left shift and megakaryocyte reduction, which was interpreted as possible evolving aplastic anemia. Family history was notable for brain abscess in his father. His father and paternal grandfather died in their late 40 s from influenza pneumonia.

**Fig. 1:**
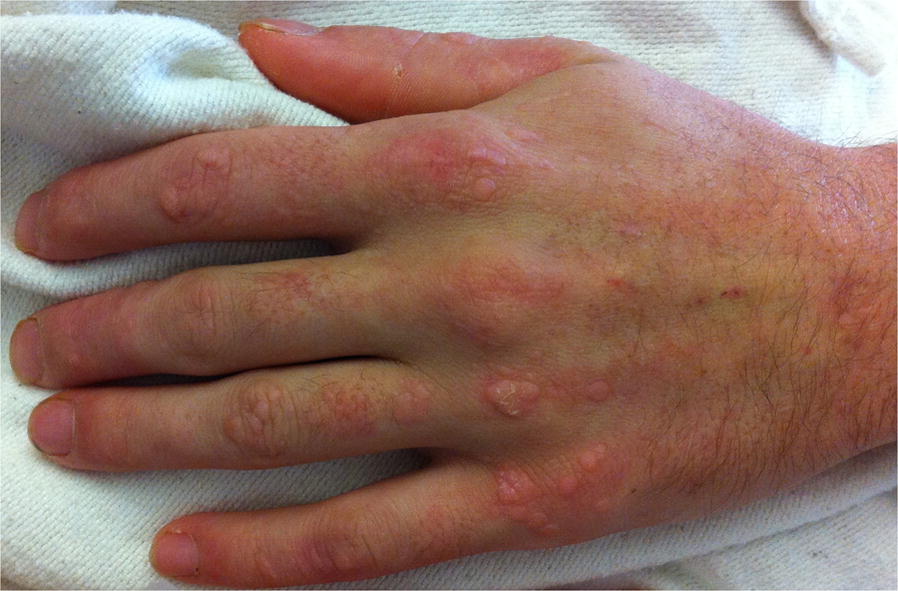
The patient was started on interferon alfa-2b. Within a few days, his warts subsided and his neurocognitive function showed significant improvement

To treat his WNVE, he received 400 mg/kg intravenous immunoglobulin (IVIG) daily for 3 consecutive days (high titer WNV immunoglobulin was not available). Based on his profile of longstanding warts due to human papillomavirus (HPV), recurrent sinopulmonary infections and lymphedema, absent circulating monocytes and significant lymphopenia, marrow hypoplasia, a paternal family history of deaths from viral illnesses, and now life-threatening WNVE, the diagnosis of GATA2 deficiency with impaired antiviral immunity was suspected. Therefore, he received 3 million units of subcutaneous interferon alfa-2b daily for 14 days [1]. Two days after starting the interferon alfa-2b, his warts and neurocognitive function improved (Fig. 1). The patient became fully cooperative and oriented, but he still had some residual poor balance, scissoring gait, and mild flu-like symptoms. The patient was discharged to a rehabilitation facility on day 14 of hospitalization and then home.

DNA sequencing showed a heterozygous single base deletion in *GATA2*, c.1021delG, causing p.A341Pfs, eliminating the highly conserved second zinc finger of GATA2. His mother did not carry the mutation, and paternal DNA was not available for sequencing. Two years after his encephalitis, he underwent a haploidentical hematopoietic stem cell transplant (HSCT) from his mother following conditioning with fludarabine, cyclophosphamide, busulfan, and total body irradiation. His course was complicated by persistent verrucae and cutaneous graft-versus-host disease.

## Discussion

The transcription factor, GATA2, is a master regulator of hematopoietic development and maintenance of the healthy stem cell pool. It recognizes the DNA motif, (A/T)GATA(A/G), using its two zinc-finger domains [2, 3]. GATA2 deficiency (also known as monocytopenia with mycobacterial disease (MonoMAC); dendritic cell (DC), myeloid, and NK cell lymphopenia (DCML); lymphedema and myelodysplasia (Emberger syndrome); and familial myelodysplasia/leukemia) is due to heterozygous germline mutations. Patients with mutations in this gene can present with highly variable phenotypes including cytopenias, myelodysplasia, acute myeloid leukemia, other hematologic and solid neoplasms, pulmonary alveolar proteinosis, thrombosis, lymphedema, sensorineural hearing loss, panniculitis, and other autoimmune disorders [3]. Recurrent infections due to viruses (such as HPV, EBV, HSV, and VZV), nontuberculous mycobacteria, and fungi are common [3].

WNVE primarily occurs in elderly and immunocompromised patients, related to reduced viral control. Our patient’s experience suggested that his genetic defect, with its associated NK, T and B lymphopenias, predisposed him to severe WNVE. Despite normal NK function in vitro, he had profound reductions in total and CD56^bright^CD16+NK cells. His NK cells were comprised almost exclusively of the CD56^dim^CD16+subset, similar to the NK cell profiles reported previously in GATA2 deficiency [4].

Use of interferon alfa-2b and IVIG had been reported in immunocompetent hosts with WNVE, some of whom subsequently recovered. However, their therapeutic roles in WNV infections are still not completely clear. Interferons are glycopeptides with immunoregulatory, antiviral, and antitumor activities with clinical applications in viral hepatitis and chronic myeloid leukemia, among other uses [5]. The addition of interferon alpha-2b in primate cell cultures before and after WNV inoculation reduced viral cytotoxicity, manifested as a recovery of cellular proliferative capacity [6]. IFN-α/β receptor-deficient mice infected with WNV also had less serum viral clearance and higher mortality, showing a possible protective role for type I interferons in WNV immunity [7]. A small study had previously suggested that immunoglobulins from donor populations with a higher prevalence of WNV exposures might contain WNV neutralizing antibody titers [8]. While the use of interferon alfa-2b and IVIG in human WNVE is limited to only a few case reports with mixed outcomes, in view of his life-threatening infection and likely underlying defect, including clearly impaired antiviral immunity as manifested by chronic HPV infection, we felt that they might have benefits [1, 9].

Currently, HSCT is the only curative option for GATA2 deficiency [10]. Case series have reported success rates ranging from ~ 50 to 86% with a median follow-up of 3.5–5 years [10]. Given the wide range of phenotypes, age at presentation and diagnosis, disease severity and rate of progression, and the risk of serious infection, as in this case, or development of leukemia, early pursuit of HSCT is reasonable. While there are variable opportunities for complete HLA matching, research is ongoing to identify the optimal conditioning and graft versus host prevention regimens for these patients.

This is the first report of a patient with GATA2 deficiency and severe WNVE. While prospective data on the management of invasive WNVE are lacking, our patient tolerated immunoglobulin and interferon alfa-2b with good clinical response.
